# Machine Learning Improves Risk Stratification in Myelodysplastic Neoplasms: An Analysis of the Spanish Group of Myelodysplastic Syndromes

**DOI:** 10.1097/HS9.0000000000000961

**Published:** 2023-10-11

**Authors:** Adrian Mosquera Orgueira, Manuel Mateo Perez Encinas, Nicolas A Diaz Varela, Elvira Mora, Marina Díaz-Beyá, María Julia Montoro, Helena Pomares, Fernando Ramos, Mar Tormo, Andres Jerez, Josep F Nomdedeu, Carlos De Miguel Sanchez, Arenillas Leonor, Paula Cárcel, Maria Teresa Cedena Romero, Blanca Xicoy, Eugenia Rivero, Rafael Andres del Orbe Barreto, Maria Diez-Campelo, Luis E. Benlloch, Davide Crucitti, David Valcárcel

**Affiliations:** 1 Complexo Hospitalario Universitario de Santiago de Compostela, Department of Hematology, Instituto de Investigacións Sanitarias de Santiago, Santiago de Compostela, Spain; 2 Hospital Central de Asturias, Oviedo, Spain; 3 Hematology Department, Hospital Universitario y Politécnico La Fe, Valencia, Spain; 4 Hospital Clinic, Dept. of Hematology, IDIBAPS, Barcelona, Spain; 5 Department of Hematology, Vall d’Hebron Institute of Oncology (VHIO), Hospital Universitari Vall d’Hebron, Barcelona, Spain; 6 Hematology Department., Hospital Duran i Reynals. Institut Català d’Oncologia, Hospital Duran i Reynals. Institut Català d’Oncologia, Hospitalet, Barcelona, Spain; 7 Department of Hematology, Hospital Universitario de León, Spain; 8 Servicio de Hematología. Hospital Clínico Universitario de Valencia, Spain; 9 Hematology and Medical Oncology Department, Hospital Morales Meseguer, IMIB, Murcia, Spain; 10 Hematology Department, Hospital de la Santa Creu i Sant Pau, Barcelona, Spain; 11 Hospital Universitario de Álava - Sede Txagorritxu, Vitoria-Gasteiz, Spain; 12 Laboratoris de Citologia Hematològica i Citogenètica, servei de Patologia, Hospital del Mar. GRETNHE- Institut Hospital del Mar d’Investigacions Mèdiques (IMIM), Barcelona, Spain; 13 Department of Hematology, Hospital Público Universitario de la Ribera, Alzira, Valencia, Spain; 14 Hospital Universitario 12 de Octubre, Instituto de Investigación Sanitaria i+12, Madrid, Spain; 15 HU German Trias i Pujol - Institut Català d’ Oncologia, Josep Carreras Leukemia Research Institute, Universitat Autònoma de Barcelona, Badalona, Spain; 16 Department of Hematology, University Hospital Arnau de Vilanova, Lleida, Spain; 17 Edif. Laboratorios, planta baja., Hospital Universitario Cruces Servicio de Hematología. Sección Eritropatología – Hem. Molecular, Barakaldo, Spain; 18 Hematology Department, Institute of Biomedical Research of Salamanca, University Hospital of Salamanca, Spain; 19 Grupo Español de Síndromes Mielodisplásicos (GESMD), Valencia, Spain; 20 Instituto de Investigacions Sanitarias de Santiago de Compostela (IDIS-CHUS), Santiago de Compostela, Spain

## Abstract

Myelodysplastic neoplasms (MDS) are a heterogeneous group of hematological stem cell disorders characterized by dysplasia, cytopenias, and increased risk of acute leukemia. As prognosis differs widely between patients, and treatment options vary from observation to allogeneic stem cell transplantation, accurate and precise disease risk prognostication is critical for decision making. With this aim, we retrieved registry data from MDS patients from 90 Spanish institutions. A total of 7202 patients were included, which were divided into a training (80%) and a test (20%) set. A machine learning technique (random survival forests) was used to model overall survival (OS) and leukemia-free survival (LFS). The optimal model was based on 8 variables (age, gender, hemoglobin, leukocyte count, platelet count, neutrophil percentage, bone marrow blast, and cytogenetic risk group). This model achieved high accuracy in predicting OS (c-indexes; 0.759 and 0.776) and LFS (c-indexes; 0.812 and 0.845). Importantly, the model was superior to the revised International Prognostic Scoring System (IPSS-R) and the age-adjusted IPSS-R. This difference persisted in different age ranges and in all evaluated disease subgroups. Finally, we validated our results in an external cohort, confirming the superiority of the Artificial Intelligence Prognostic Scoring System for MDS (AIPSS-MDS) over the IPSS-R, and achieving a similar performance as the molecular IPSS. In conclusion, the AIPSS-MDS score is a new prognostic model based exclusively on traditional clinical, hematological, and cytogenetic variables. AIPSS-MDS has a high prognostic accuracy in predicting survival in MDS patients, outperforming other well-established risk-scoring systems.

## Graphical abstract

**Figure:**
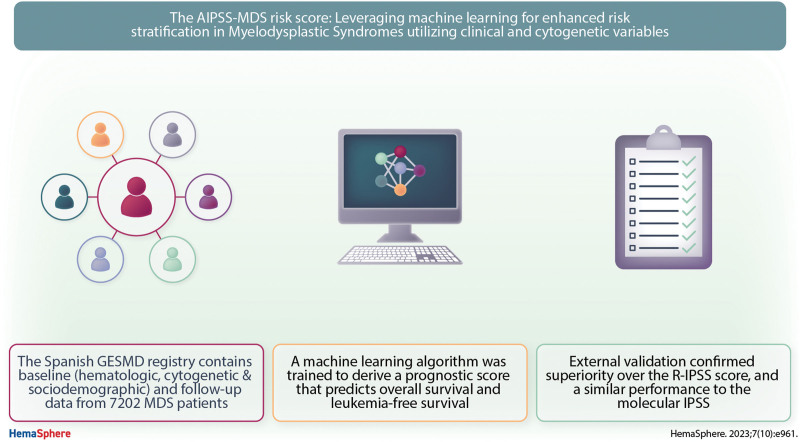

## INTRODUCTION

Myelodysplastic neoplasms (MDS) comprise a variety of diagnostic entities characterized by the presence of dysplasia, cytopenias and risk of progression to bone marrow (BM) failure or acute myeloid leukemia.^1^ Currently, MDS are classified according to the presence of defining genetic abnormalities or the observation of morphological dysplasia.^2^ MDS exhibit remarkably different clinical phenotypes and prognosis, which is related to its inherent genomic complexity. Driver mutations affect genes involved in diverse pathways, such as DNA methylation, gene expression regulation, chromatin modification, signal transduction, and mRNA splicing.^3,4^ The presence and interaction of these different driver mutations dictate the evolutionary trajectory of MDS, and are therefore correlated with clinical phenotypes and disease prognosis.^3,5^ Therefore, clinical and laboratory parameters at MDS diagnosis resume the underlying molecular background.

Currently, the only curative treatment for MDS is allogeneic hematopoietic stem cell transplantation (allo-HCT), but it is reserved for a minority of fit patients due to its significant morbidity and mortality.^6,7^ Furthermore, the variable availability of suitable donors and the substantial economic burden associated with the procedure pose significant challenges that need to be addressed for broader accessibility and improved patient outcomes. Improving risk prediction in MDS is key to selecting optimal candidates for allo-HCT, facilitating a balanced decision between toxicity and disease severity. To date, the Revised International Prognostic System (IPSS-R) is the standard method to determine patient risk of progression to AML and death.^8,9^ This score considers 5 variables: hemoglobin, absolute neutrophil count, platelet count, BM blasts and cytogenetic risk category, and it assigns patients to 5 asymmetric risk groups. Patients with very low and low-risk disease have a prolonged survival (median overall survival [OS] of 8.8 and 5.3 years, respectively), intermediate-risk patients have a median OS of 3 years, whereas high and very high-risk patients have an adverse prognosis (median OS of 1.6 and 0.8 years, respectively). A variation of the IPSS-R score which adjusts survival for patient age (IPSS-RA) was also developed, but the use of this score has not been so extended as that of the IPSS-R.

Recently, a new proposal for risk stratification in MDS using molecular data has been proposed (molecular IPSS; IPSS-Mol), which incorporates mutations in 31 genes into the prognostic system.^5^ This model achieved a higher discriminative capacity compared with the IPSS-R (IPSS-Mol c-index; 0.75; IPSS-R c-index; 0.70), and it is expected to become the new gold standard. However, there are some issues related to genomic data that need to be evaluated. First, genomic analysis is complex and inaccessible to most patients in low and middle-income countries, becoming a source of increasing inequality in healthcare delivery.^10^ Second, it has been observed that the prognostic impact of somatic mutations involving some drivers of myelodysplasia is heterogeneous, with conflicting interpretations and further refinements in the recent literature. For example, Bernard et al discovered that monoallelic *TP53* hits did not influence prognosis in MDS patients, whereas multihit somatic events were independently associated with adverse outcome.^11^ Results by Montalban-Bravo et al (2020) indicate that the prognostic role of *TP53* is not only influenced by multihit mutations but also by variant allele frequency and genomic context.^12^ More recently, Weiberg et al (2022) observed an adverse impact of *TP53* disruption (both as monoallelic or multihit events); and no additional prognostic impact was found neither for *TP53* variant allele frequency nor for co-occurring somatic mutations.^13^ In a different case, although MDS with *SF3B1* mutation has been recognized as a different disease subgroup characterized by a relatively indolent prognosis, recent data indicate that the prognostic role of *SF3B1* is substantially conditioned by the type of mutation and genomic diversity.^5,14,15^ Therefore, although the IPSS-Mol advances over previous classifications, some caveats exist about both its logistical implementation and the degree of biological variation used to develop the model.

Improving prognostic systems using basic information has the potential to rapidly impact the field by enabling fast risk stratification in most healthcare facilities, which can facilitate treatment choice. The recent development of machine learning (ML) in medicine has become key to overcoming some of the limitations of classical prognostic scores.^16,17^ ML is a field of artificial intelligence in which an algorithm creates predictions that are based on a learning phase from real examples, instead of depending on human-made rules. In ML, modeling occurs by considering simple and complex interactions between multiple variables. In the case of MDS, these advanced techniques can provide personalized survival predictions based on the clinical outcomes of thousands of patients. With this in mind, we aimed to develop a new ML model for MDS risk stratification using traditional variables obtained at disease diagnosis.

## MATERIALS AND METHODS

### Data source

We retrieved original data included in the Spanish Registry of Myelodysplastic Syndromes, which comprised 7,202 patients diagnosed with MDS in 90 centers between May 29, 2006 and January 15, 2022. This is a nationwide registry (code: 2018/0459) contributed by centers affiliated to the *Grupo Español de Síndromes Mielodisplásicos* (GESMD). Informed consent for inclusion in the registry was obtained from all patients. MDS diagnosis was made according to the World Health Organization 2008 classification. The study was approved by the GESMD scientific board and was conducted in accordance with the Declaration of Helsinki.

The database included 18 variables with a missing rate of <50%. The variables were: age at diagnosis, gender, proportion of blasts in peripheral blood, proportion of blasts in BM, serum erythropoietin, proportion of nucleated red cells in BM, proportion of ring sideroblasts in BM, presence of Auer rods in BM, cellularity in BM smear (hypocellular, normocellular and hypercellular), serum lactate dehydrogenase, serum ferritin, hemoglobin, leukocytes, platelets, absolute neutrophil count, the proportion of neutrophils, proportion of monocytes and cytogenetic risk group according to the IPSS-R. Patients were randomly divided into a training set (80% of the cohort, N= 5760) and a test set (20% of the cohort, N= 1442). Univariable analysis (cox regression) was used to evaluate the relationship of these variables with clinical outcomes.

### Main study outcomes

OS was defined as time from MDS diagnosis to death from any cause. Acute leukemia-free survival (LFS) was defined as time from MDS diagnosis to date of leukemic transformation (≥20% blasts in BM or peripheral blood) or last contact/date of death.

### Variable selection and model development

Univariable Cox regression was used to evaluate the association of each variable with OS in the training set (survival package, version 3.5.3).^18^ Variables with a *P*-value <0.05 were selected for a multivariable model using random survival forests (RSF, randomForestSRC package, version 3.2.0).^19^ The use of random forests was based on the tabular nature of our initial data. Despite claims of superior performance by deep learning models, evidence indicates that decision tree-based models consistently outperform deep learning across various fields.^20^ RSF models were created with 1000 trees. Missing variables were imputed using a missing data algorithm developed by Ishawarian et al.^19^ Predictions were cross-validated in the training cohort and then validated in the test cohort. For cross-validation, sampling was performed without replacement, which by default takes 0.632 times the sample size. This was done in order to rule out overfitting of performance metrics in the training set related to either variable selection or the imputation process. The discriminative capacity of the RSF models in the training set was evaluated with out-of-bag estimates of the concordance index (c-index).

A dynamic assessment of the different predictors was performed using time-dependent areas under the curve (AUCs) derived from Cox survival models (*riskRegression* package, version 2021.10.10).^21^ Time-dependent Brier scores, a measure of calibration and accuracy, were calculated as the mean squared difference between the predicted probability and the actual outcomes. For these calculations, cross-validation was performed using 500 cycles. In each cycle, 75% of samples were used for training and 25% for testing. The c-indexes of these Cox models were computed with bootstrapping in both the training and test sets (500 cycles). In the particular case of the training set, all RSF predictions used as input for downstream analysis were out-of-bag to reduce the risk of overfitting during the training phase of the model. By utilizing the 3 different metrics, we aim to provide a comprehensive evaluation of our model’s performance, covering various aspects of its predictive abilities. Each metric offers unique information that informs about different aspects of the model’s performance: the C index assesses ranking and discrimination, the Brier score evaluates calibration and accuracy, and the time-dependent AUC accounts for dynamic discriminatory ability.

### External validation

Data from the cohort by Bernard et al (2022) was downloaded from their public GitHub repository. Patients with complete annotations for the 8 and 10-variable AIPSS-MDS models were selected. The survival function based on data from the Spanish cohort was calculated in each of these patients, and the accuracy of the prediction was calculated using the c-index. For comparison with the IPSS-R and IPSS-Mol scores, we calculated the AIPSS-MDS score as the result of the cumulative hazard function for each patient. Finally, the comparisons between the 3 scores were based on cross-validated AUCs of the Cox models.

### Data sharing

For original dataset sharing, please contact the following email: adrian.mosquera.orgeira@sergas.es.

## RESULTS

### Study population, clinical outcomes, and variable selection

The baseline characteristics and main clinical outcomes of each cohort are presented in Table 1. Median follow-up was 4.93 years and 5.20 years in the training and test sets, respectively. 26.11% and 24.46% of patients were treated with a disease-modifying therapy (demethylating drugs, immunomodulatory imid drugs, chemotherapy, or allo-HCT). during follow-up. Seven variables had a missing rate >10% in both cohorts, as indicated in Suppl. Table S1. Fifteen variables were associated with LFS with a *P*-value ≤0.05 in the training set, and 17 variables did so with OS (Table 2).

**Table 1:** Basal Characteristics of the Training and Test Cohorts

	Training Set	Test Set
N	5760	1442
Median age	75 years	75 years
Gender	59% male/41% female	58% male/42% female
Median BM blasts	2%	2%
Median blasts in peripheral blood	0%	0%
Median Hb	9.9 g/dL, IQR 2.5	9.9 g/dL, IQR 2.5
Median platelets	149.5 × 10^9^/L, IQR 170	157.0 × 10^9^/L, IQR 176
Median PMN	1.98 × 10^6^/L, IQR: 2.29	1.94 × 10^6^/L, IQR 2.22
Median leucocytes	4.09 × 10^6^/L, IQR 3.1	4.00 × 10^6^/L, IQR 3.0
Very good cytogenetics	4.20%	4.44%
Good cytogenetics	70.7%	68.8%
Intermediate risk cytogenetics	10.3%	11.3%
Poor cytogenetics	3.7%	3.9%
Very poor cytogenetics	5.8%	5.8%
IPSS-R very low	25.5%	24.3%
IPSS-R low	38.1%	41.1%
IPSS-R intermediate	17.6%	15.7%
IPSS-R high	10.4%	9.7%
IPSS-R very high	8.4%	9.2%
Median follow-up	4.9 years, IQR 7,3	5.2 years, IQR 7,2
Median OS	4.4 years, IQR 7,2	4.4 years, IQR 7,3
5q- MDS	5.4%	5.7%
MDS-ULD	9.4%	8.8%
MDS-MLD	43.2%	44.7%
RARS	11.8%	11.5%
EB-1	15.7%	14.5%
EB-2	13.9%	14.0%
MDS-unclassified	0.7%	0.8%
AML transformation	10.8%	9.7%
Treated with demethylating drugs	18.95%	17.16%
Treated with IMIDs	6.32%	2.95%
Treated with chemotherapy	0.76%	0.42%
Performed Allo-HCT	4.5%	4.5%

IPSS-Mol = molecular International Prognostic Scoring System; IPSS-R = revised International Prognostic Scoring System; IQR = interquartile range; MDS = myelodysplastic neoplasm; MDSMLD = myelodysplastic neoplasm with multilineage dysplasia; MDS-ULD = myelodysplastic neoplasm with unilineage dysplasia; OS = overall survival; RARS = Refractory Anemia with Ring Sideroblast..

**Table 2:** Association of the Different Variables With LFS and OS in the Training Set (Univariable Cox *P*-value)

Variable	OS *P*-value and HR (95% CI)	Leukemia-free Survival *P*-value and HR (95% CI)
Age	<0.0001, 1.03 (1.026–1.033)	<0.0001, 0.98 (0.98–0.99)
Gender (female vs male)	<0.0001, 1.34 (1.24–1.44)	0.0084, 1.25 (1.06–1.47)
Peripheral blood blasts (%)	<0.0001, 1.14 (1.12–1.16)	<0.0001, 1.23 (1.21–1.26)
Bone marrow blasts (%)	<0.0001, 1.10 (1.09–1.10)	<0.0001, 1.18 (1.17–1.20)
Absolute value of red nucleated cells in bone marrow smear (%)	0.005, 1.00 (1.00–1.01)	<0.0001, 1.00 (1.00–1.01)
Presence of Auer rods (no vs yes)	0.003, 1.75 (1.22–2.49)	<0.0001, 5.25 (3.27–8.42)
Ring sideroblasts (%)	<0.0001, 0.99 (0.99–0.99)	<0.0001, 0.98 (0.97–0.98)
Bone marrow cellularity (hypercellular vs normal)	0.002, 0.86 (0.79–0.94)	0.06, 0.87 (0.72–1.05)
Bone marrow cellularity (hypercellular vs hypocellular)	0.07, 1.05 (0.92–1.19)	0.15, 1.29 (0.99–1.67)
LDH (units/µL)	<0.0001, 1.00 (1.00–1.01)	<0.0001, 1.00 (1.00–1.01)
Serum erythropoietin	<0.0001, 0.99 (0.99–1.00)	0.05, 0.98 (0.98–0.99)
Free transferrin light chain	<0.0001, 1.00 (1.00–1.01)	0.0005, 1.00 (1.00–1.01)
Hemoglobin (g/dL)	<0.0001, 0.82 (0.81–0.83)	<0.0001, 0.88 (0.84–0.91)
Leukocytes	0.007, 0.98 (0.96–0.99)	<0.0001, 0.93 (0.90–0.07)
Platelets	<0.0001, 0.99 (0.99–0.99)	<0.0001, 0.99 (0.99–0.99)
Neutrophil count	0.04, 0.98 (0.96–0.99)	<0.0001, 0.86 (0.81–0.91)
Neutrophil %	<0.0001, 0.99 (0.99–0.99)	<0.0001, 0.97 (0.97–0.98)
Monocyte %	0.02, 0.99 (0.98–0.99)	0.03, 0.98 (0.97–0.99)

CI = confidence interval; LDH = lactate dehydrogenase; LFS = leukemia-free survival.

### Prediction of OS and leukemic transformation

**Figure 1.**
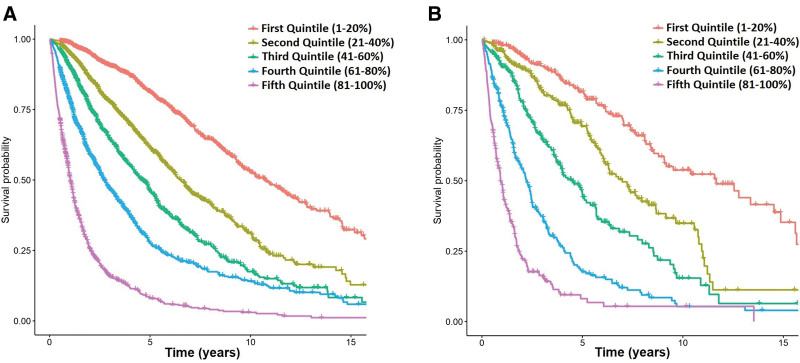
**Representation of OS for patients in the training (A) and test (B) sets according to the different quintiles of expected survival predicted by the ML model.** ML = machine learning; OS = overall survival.

An RSF model was created to predict OS using the significant variables identified in the training set. This model achieved a c-index of 0.765 in the training set and 0.782 in the test set (Table 3). The c-indexes of the IPSS-R predictor considering RSF were 0.656 and 0.705 in the training and test set, respectively (Suppl. Figure S1). Seven variables with >10% missing rate were discarded, along with the absolute neutrophil count due to its less significant association with OS, resulting in a 10-variable model, which achieved c-indexes of 0.760 and 0.778 in the training and test set, respectively (Suppl. Table S2 and Suppl. Table S3). Finally, we reasoned that the proportion of blasts in peripheral blood was redundant with the proportion of blasts in BM. We also observed that the association of the proportion of monocytes with OS was relatively weak (Table 2). Therefore, a model containing 8 variables was constructed which achieved c-indexes of 0.759 and 0.776 in the training and test set (Suppl. Table S2 and Suppl. Table S3). Due to its reduced dimensionality and similar performance to the other models, we chose the 8-variable model for further evaluation (Figure 1 and Suppl. Table S3). This model included the following baseline variables: age, sex, percentage of BM blasts, hemoglobin levels, platelet and leukocyte counts, percentage of neutrophils, and cytogenetic risk group according to the IPSS-R classification. We also studied the performance of the IPSS-R-related variables as input to the RSF model, revealing a better performance compared with the IPSS-R (c-indexes; 0.736 and 0.746 in the training and test sets), but still inferior to the new model.

**Table 3:** C-indexes and 95% CI of the Different ML Models for OS Prediction

C-index	Training Set	Test Set
ML1 (18 variables)	0.765	0.782
ML2 (10 variables)	0.760	0.778
ML3 (8 variables)	0.759	0.776

ML = machine learning; OS = overall survival.

**Figure 2.**
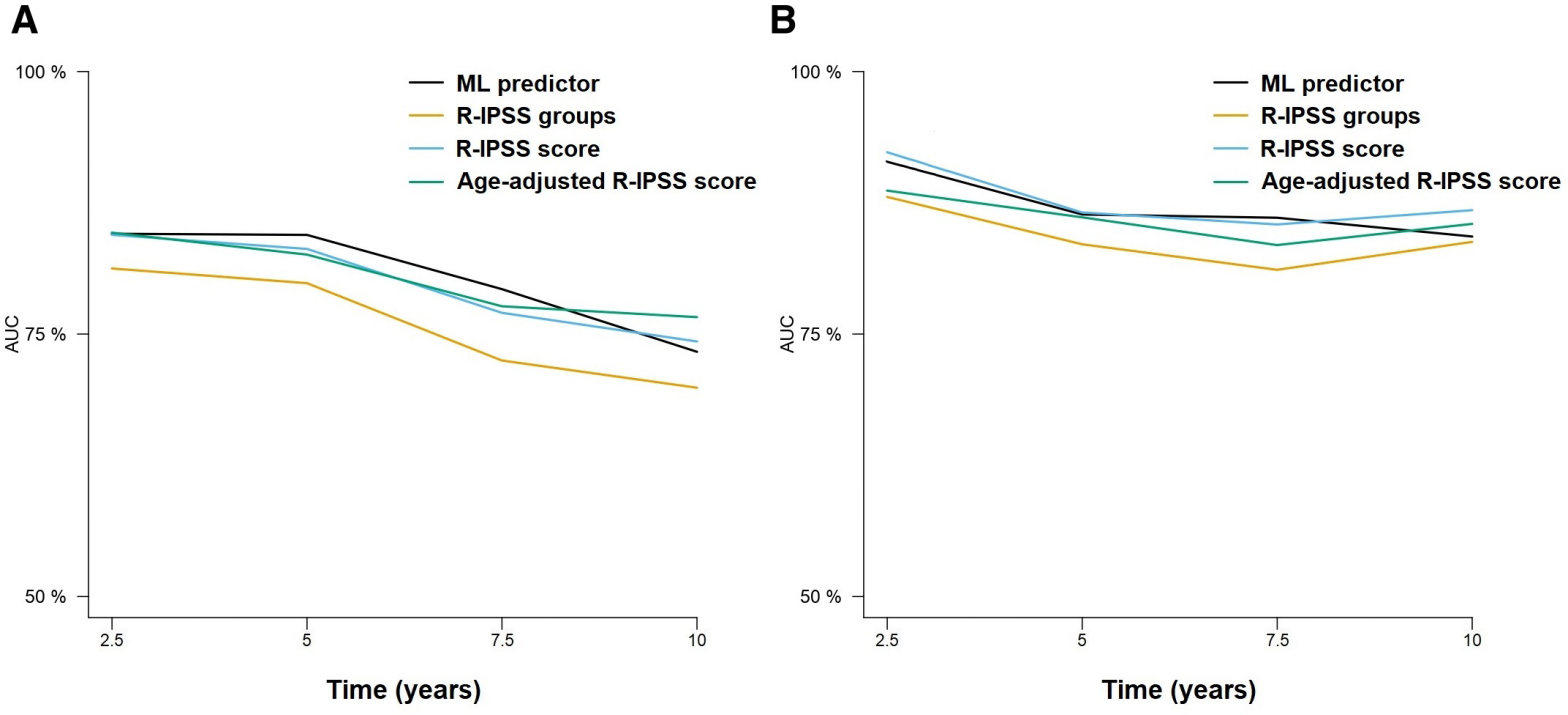
**Time-dependent AUCs at 2.5, 5, 7.5, and 10 years after diagnosis of the different prognostic models for the prediction of LFS.** Results for the training (A) and test (B) sets are represented. Only the 8-variable ML model is represented for the sake of simplicity. Results for the training (A) and test (B) sets are represented. Only the 8-variable ML model is represented for the sake of simplicity. AUCs = areas under the curve; LFS = leukemia-free survival; ML = machine learning.

Then, we tested the 10 and 8-variable models to test time to AML transformation using RFS. C-indexes of the 10-variable model were 0.816 and 0.846 in the training and test sets, whereas the c-indexes of the 8-variable model were 0.812 and 0.845 in the training and test sets, respectively (Suppl. Table S2). In comparison, the IPSS-R score achieved c-indexes of 0.715 and 0.807 in the training and test sets. Time-dependent cross-validated AUCs also indicated that the new predictor achieved high accuracy (Figure 2), which was superior to the IPSS-R groups and similar to the performance of the numeric IPSS-R score.

### Comparison of the ML model with the IPSS-R for predicting OS

**Figure 3.**
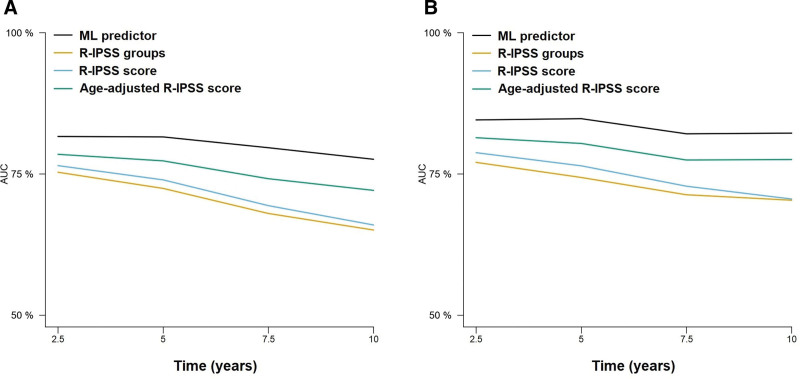
**Time-dependent AUCs at 2.5, 5, 7.5, and 10 years after diagnosis of the different prognostic models for the prediction of OS.** Results for the training (A) and test (B) sets are represented. Results for the training (A) and test (B) sets are represented. AUCs = areas under the curve; OS = overall survival.

The performance of the RSF model was assessed using cross-validated time-dependent AUCs, and compared with those of the IPSS-R and the IPSS-RA. The ML model achieved a higher AUC and higher Brier scores compared with the IPSS-R and IPSS-RA, in both the training and test cohorts (Figure 3, Table 4, and Suppl. Table S4). Moreover, we also observed that the RSF model provided superior results compared with Cox regression, particularly in the prediction of 5-year mortality. Furthermore, we were able to confirm the superiority of the new model over the IPSS-R in patients under and over 65 years (Suppl. Figure S2 and Suppl. Table S5).

**Table 4:** Time-dependent AUCs for the Different OS Predictors in the Training and Test Sets

Model	Times	Training Set AUC	Test Set AUC
ML predictor—18 variables	2.5	00823 (0.802–0.844)	0.852 (0.813–0.894)
ML predictor—18 variables	5	0.816 (0.791–0.839)	0.854 (0.809–0.896)
ML predictor—18 variables	7.5	0.793 (0.759–0.823)	0.821 (0.755–0.877)
ML predictor—18 variables	10	0.769 (0.714–0.811)	0.805 (0.703–0.898)
ML predictor—10 variables	2.5	0.818 (0.796–0.840)	0.848 (0.809–0.890)
ML predictor—10 variables	5	0.815 (0.791–0.838)	0.851 (0.808–0.891)
ML predictor—10 variables	7.5	0.796 (0.764–0.826)	0.823 (0.757–0.880)
ML predictor—10 variables	10	0.775 (0.722–0.817)	0.818 (0.722–0.909)
ML predictor—8 variables	2.5	0.816 (0.795–0.834)	0.846 (0.804–0.886)
ML predictor—8 variables	5	0.816 (0.791–0.838)	0.848 (0.801–0.889)
ML predictor—8 variables	7.5	0.797 (0.764–0.825)	0.821 (0.757–0.876)
ML predictor—8 variables	10	0.776 (0.725–0.819)	0.822 (0.735–0.908)
Cox-PH—8 variables	2.5	0.797 (0.772–0.821)	0.819 (0.777–0.861)
Cox-PH—8 variables	5	0.805 (0.782–0.831)	0.837 (0.789–0.876)
Cox-PH—8 variables	7.5	0.800 (0.769–0.832)	0.817 (0.754–0.874)
Cox-PH—8 variables	10	0.800 (0.750–0.841)	0.834 (0.753–0.915)
IPSS-R groups	2.5	0.753 (0.728–0.778)	0.771 (0.714–0.821)
IPSS-R groups	5	0.724 (0.695–0.754)	0.744 (0.693–0.795)
IPSS-R groups	7.5	0.680 (0.645–0.715)	0.713 (0.645–0.776)
IPSS-R groups	10	0.651 (0.600–0.701)	0.704 (0.597–0.810)
IPSS-R numeric score	2.5	0.765 (0.740–0.790)	0.788 (0.735–0.839)
IPSS-R numeric score	5	0.740 (0.710–0.769)	0.764 (0.709–0.815)
IPSS-R numeric score	7.5	0.694 (0.657–0.731)	0.728 (0.650–0.794)
IPSS-R numeric score	10	0.660 (0.607–0.773)	0.706 (0.600–0.616)
IPSS-RA numeric score	2.5	0.785 (0.760–0.810)	0.814 (0.767–0.859)
IPSS-RA numeric score	5	0.773 (0.748–0.801)	0.804 (0.755–0.853)
IPSS-RA numeric score	7.5	0.742 (0.705–0.777)	0.775 (0.702–0.841)
IPSS-RA numeric score	10	0.721 (0.665–0.773)	0.776 (0.669–0.881)

AUCs = areas under the curve; IPSS-R = revised International Prognostic Scoring System; ML = machine learning; OS = overall survival..

**Figure 4.**
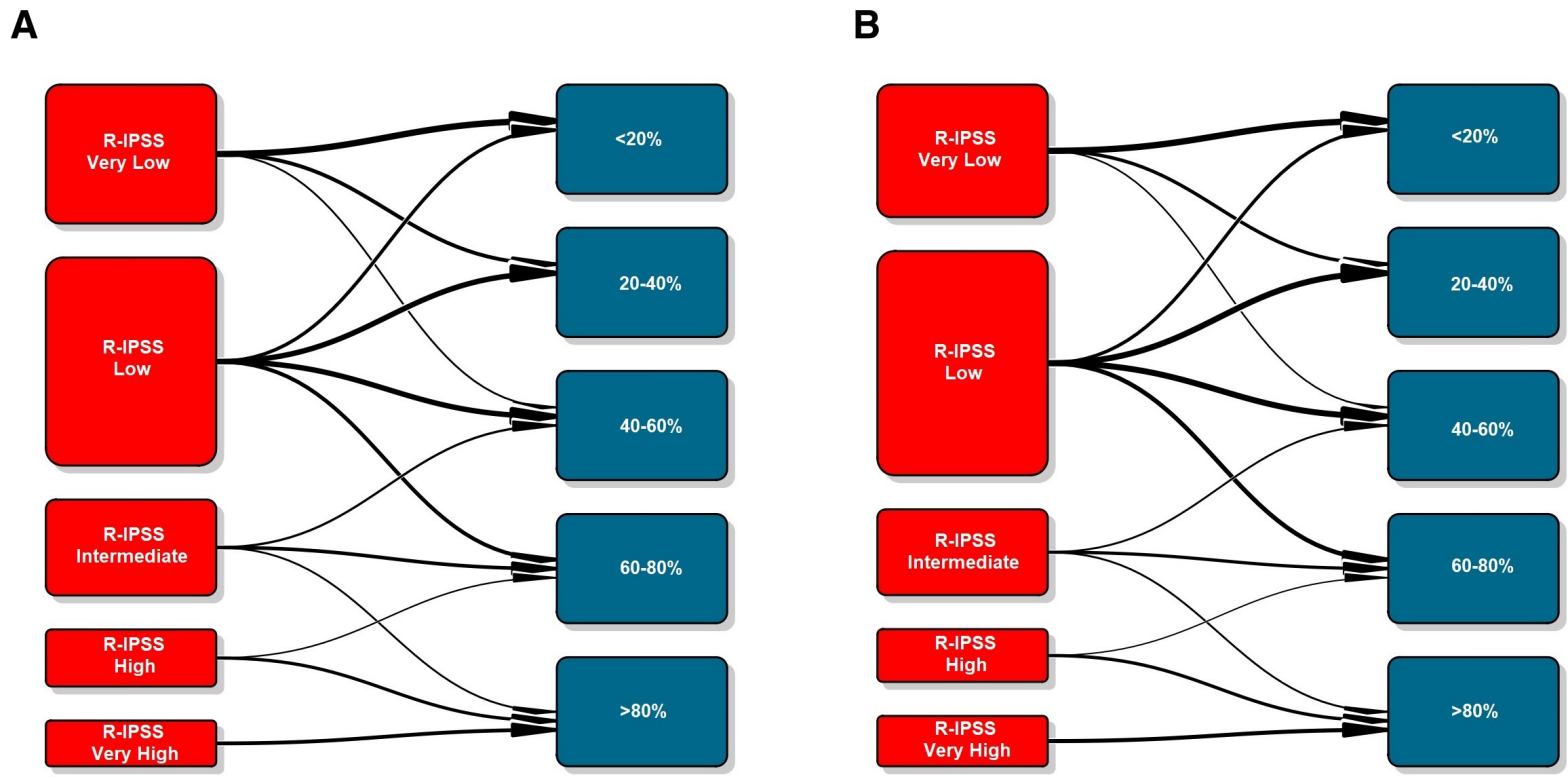
**Transition plots between IPSS-R groups and the quintiles of expected OS predicted by the ML model in the training (A) and test (B) sets.** IPSS-R = revised International Prognostic Scoring System; ML = machine learning; OS = overall survival.

To get a graphical perspective about the performance of the AIPSS-MDS in comparison with the IPSS-R score, we clusterized MDS patients in 5 equally-sized risk groups according to the predicted risk. Next, the IPSS-R groups were compared with these quintiles of risk predicted. The distribution of the IPSS-R groups was unbalanced, with only 8.54% and 10.29% of patients being assigned to a very high and high-risk group. On the contrary, as the distribution of patients by the RSF algorithm is continuous, these could be represented in balanced groups (Table 1 and Figure 4). The comparison revealed that 60.97% and 60.07% of patients from the training and test set were assigned to a different risk group than that assigned by the IPSS-R, a fact which was particularly relevant among low, intermediate, and high-risk groups.

### Performance analysis in relevant diagnostic subgroups

The algorithm outperformed the numeric IPSS-R and the IPSS-RA in the prediction of 5-year mortality in all MDS subtypes, including MDS with unilineage dysplasia, MDS with multilineage dysplasia, MDS with Excess Blasts type 1 (MDS-EB-1), MDS with Excess Blasts type 2 (MDS-EB-2), Refractory Anemia with Ring Sideroblasts and MDS with isolated 5q deletion (Suppl. Figure S3, Suppl. Figure S4, Suppl. Figure S5, Suppl. Figure S6, Suppl. Figure S7, Suppl. Figure S8, and Suppl. Table S6). Importantly, 5-year survival predictions made by the ML model in the test set were superior to the remaining models in all diagnostic subgroups, with particularly high differences observed in the case of MDS-EB-1, MDS-EB-2, and MDS with isolated 5q deletion.

### External validation of the model and comparison with the IPSS-Mol

The cohort data published by Bernard et al (2022) was retrieved from a public repository.^5^ Overall, the database contained data from 2957 patients. In all those cases with missing annotations in any of the variables used to construct the AIPSS-MDS model, the IPSS-R score or the IPSS-Mol score were removed. This resulted in 1548 patients for the analysis of OS and 1427 patients for the analysis of LFS.

**Figure 5.**
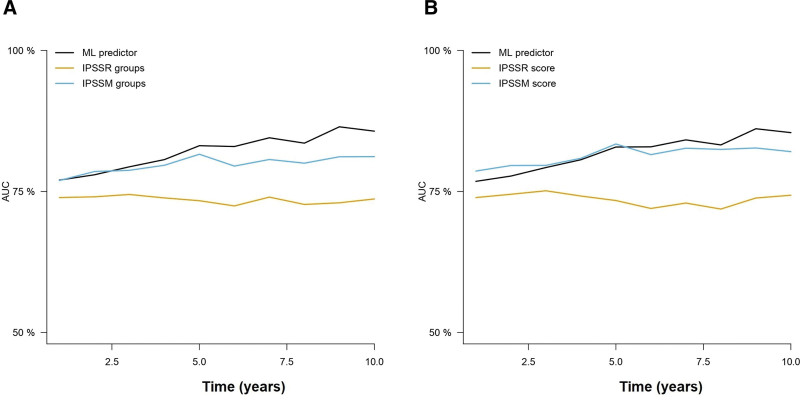
**Time-dependent AUCs of the different cross-validated Cox models comparing the AIPSS-MDS, IPSS-R, and IPSS-Mol models for OS prediction.** A) The AIPSS-MDS quantitative core is compared with the IPSS-R and IPSS-Mol groups. B) The AIPSS-MDS quantitative core is compared with the IPSS-R and IPSS-Mol quantitative scores. AIPSS-MDS = Artificial Intelligence Prognostic Scoring System for myelodysplastic neoplasm; AUCs = areas under the curve; IPSS-Mol = molecular International Prognostic Scoring System; IPSS-R = revised International Prognostic Scoring System; OS = overall survival.

For OS prediction, the RFS model trained with 8 and 10 variables in the Spanish cohort achieved c-indexes of 0.734 and 0.735 in the cohort by Bernard et al (2022).^5^ Then, the cumulative hazard scores were retrieved for comparison with the IPSS-R and IPSS-Mol scores in this group of patients. The results of Cox regression models suggested a similar performance between the AIPSS-MDS models and the IPSS-Mol, with an apparent advantage of the IPSS-Mol in terms of c-index within the standard error margin with the AIPSS-MDS model (Table 5). To evaluate if this difference was derived from overfitting of the IPSS-Mol to its original training set, cross-validated AUCs were computed and plotted (Figure 5 and Suppl. Table S7). The findings confirmed the superiority of the AIPSS-MDS score over the IPSS-R grouping and scoring systems. More importantly, the results of the AIPSS-MDS were similar to those of the IPSS-Mol score and superior to the IPSS-Mol grouping strategy.

**Table 5:** C-indexes of the Cox Models Based on the AIPSS-MDS, IPSS-R, and IPSS-Mol Scores in the Cohort by Bernard et al (2022)^5^

	OS C-index (s.e.)	R2 Value	LFS C-index (s.e.)	R2 Value
8-variable ML model	0.735 (0.01)	0.23	0.804 (0.01)	0.14
10-variable ML model	0.736 (0.01)	0.24	0.801 (0.01)	0.13
IPSS-R groups	0.700 (0.01)	0.18	0.766 (0.02)	0.13
IPSS-M groups	0.735 (0.01)	0.24	0.804 (0.01)	0.18
IPSS-R score	0.704 (0.01)	0.18	0.782 (0.02)	0.12
IPSS-M score	0.749 (0.01)	0.26	0.822 (0.01)	0.19

AIPSS-MDS = Artificial Intelligence Prognostic Scoring System for myelodysplastic neoplasm; IPSS-Mol = molecular International Prognostic Scoring System; IPSS-R = revised International Prognostic Scoring System; LFS = leukemia-free survival; ML = machine learning; OS = overall survival; s.e. = standard error..

**Figure 6.**
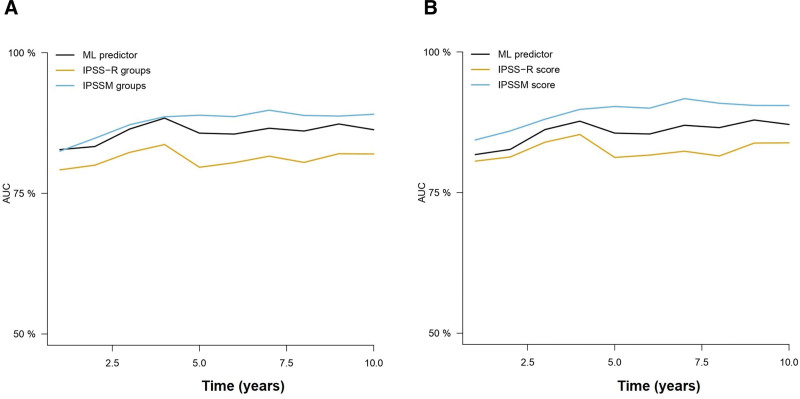
**Time-dependent AUCs of the different cross-validated Cox models comparing the AIPSS-MDS, IPSS-R, and IPSS-Mol models for LFS prediction.** (A) The AIPSS-MDS quantitative core is compared with the IPSS-R and IPSS-Mol groups. (B) The AIPSS-MDS quantitative core is compared with the IPSS-R and IPSS-Mol quantitative scores. AIPSS-MDS = Artificial Intelligence Prognostic Scoring System for myelodysplastic neoplasm; AUCs = areas under the curve; IPSS-Mol = molecular International Prognostic Scoring System; IPSS-R = revised International Prognostic Scoring System; LFS = leukemia-free survival.

Similarly, we used the LFS model trained in the Spanish cohort to derive LFS risk estimates. The results of these predictions rendered c-indexes of 0.804 and 0.801 using the 8 and 10-variable AIPSS-MDS model. Cox regression results suggested a similar performance between the IPSS-Mol grouping and the AIPSS-MDS models, but a slightly better result for the IPSS-Mol quantitative scores. Using cross-validated Cox regression, we confirmed the superiority of the AIPSS-MDS score over both the IPSS-R (Figure 6 and Suppl. Table S7). In this case, the performance of the AIPSS-MDS was similar to that of the IPSS-Mol grouping strategy but slightly inferior to that of the IPSS-Mol quantitative scores.

## DISCUSSION

In the present study, we created a ML model to predict overall and LFS in MDS based on a large cohort of patients developed by the Spanish Myelodysplastic Syndromes Group (GESMD). As a result, we constructed a supervised ML model based on an RSF algorithm which comprised 8 variables obtained at the time of MDS diagnosis. The Artificial Intelligence Prognostic Scoring System for MDS (AIPSS-MDS) model outperforms currently established prognostic systems, such as the IPSS-R and the IPSS-RA. In addition, the AIPSS-MDS model produces balanced risk groups, reclassifying roughly 60% of the patients into a different risk category compared with the IPSS-R. Other important advantages of the new model are that it provides a personalized risk estimate for each individual patient; and that it is not based on genomic data, enabling a rapid implementation in most types of healthcare facilities.

Due to the remarkable clinical heterogeneity of MDS, an optimal risk stratification is key for treatment decision, particularly for those fit patients who could benefit from allo-HCT. The IPSS-R score has been the gold standard method until now, but evidence indicates a marginal improvement in discriminative power for OS and LFS prediction compared with other methods such as the IPSS and the WHO Classification-based Prognostic Scoring System.^22^ Furthermore, the performance of the IPSS-R in some groups of patients appears to be suboptimal. For example, data from the European LeukemiaNet indicate that the IPSS and IPSS-R have a modest performance in the prediction of OS among low-risk MDS patients, with substantial improvements provided by the IPSS-RA.^23^ An interesting approach to improve over these scores was presented by Nazha et al, who used ML tools to incorporate both traditional information and NGS data into the risk stratification. This resulted in a model which achieved greater discriminative power for both OS and AML transformation prediction than the IPSS and the IPSS-R scores.^24^ More recently, the IPSS-Mol has been proposed, which incorporates the mutation status of 31 prognostic genes, hemoglobin level, platelet count, BM blasts, and the IPSS-R cytogenetic group.^5^ The score was designed as a weighted sum of the prognostic variables and finally categorized patients into 6 risk groups. This model outperformed the prognostic capacity of the IPSS-R model, reclassifying up to 44% of the patients. The comparison of the AIPSS-MDS with the IPSS-Mol revealed a similar performance of the 2 scores for OS prediction, with the AIPSS-MDS score strategy being moderately superior to the IPSS-Mol grouping and similar to the IPSS-Mol quantitative score. With regard to the prediction of LFS, the AIPSS-MDS tended to be slightly inferior to the IPSS-Mol in both cases. Nevertheless, it should be noted that this cohort was the same used to construct the IPSS-Mol score, and some overfitting of this model to the population characteristics could be expected. Therefore, we believe that the head-to-head comparison of the 2 scores in other independent cohorts will shed new light about the real performance of the IPSS-Mol with respect to the AIPSS-MDS system.

Four main factors drive the good performance of the AIPSS-MDS model. First, there is a widely known correlation between somatic mutations and clinical phenotypes. Therefore, the variability explained by hematological parameters and classical cytogenetics partially overlaps with the information derived from the mutational profile.^25^ Second, we included some new features associated with clinical outcomes, such as age and gender, which have been previously suggested to be used for refining risk scores in MDS.^26^ Third, we substituted absolute neutrophil counts by relative counts, as these were more informative according to our data, a finding which was also reported during the development of the IPSS-Mol.^5^ Finally, we used an ML tool trained in a high number of patient cases, integrating their information into a simplified survival prediction for each new patient, instead of classifying patients into closed-risk groups. The improved prognostication of the AIPSS-MDS affected most of the patients, with up to 60% of them being reclassified to a different risk level. Such improvement was related to the ability of the AIPSS-MDS to redefine the very low, low, intermediate, and high-risk categories of the IPSS-R classification. In the future, the addition of mutational profiles and frailty classifications to this score will be studied to evaluate further improvements in risk stratification.

The comprehensive evaluation of our proposed risk assessment tool by the wider community of treating physicians should be encouraged. By comparing its performance with established scores, such as IPSS-R and IPSS-M, we can gain valuable insights into its clinical utility and potential benefits for prognostication and treatment decisions across different chronic myeloid malignancies and in multiple geographical regions. As we recognize the parallel use of multiple prognosis scores in other disease contexts, our model presents one among many solutions. Nonetheless, the principal advantage of the present prognostic model relies on its capacity to accurately predict MDS prognosis without the need for complex genomic data. We emphasize that our approach is specifically designed to cater to patient cohorts in less developed countries, where access to complex genomic data might be limited. By focusing on essential clinical parameters, such as cytomorphology, peripheral blood count, and cytogenetics, our web-based calculator provides a valuable risk stratification tool that can significantly benefit many patients in resource-constrained settings. While we acknowledge that IPSS-M might represent the future of risk assessment, the present calculator serves as a practical solution for those without access to advanced genomic tools, helping to bridge the gap in risk prediction and ensuring equitable healthcare outcomes for diverse socioeconomic and geographical populations. In this line, it has been proposed that the incorporation of NGS in clinical practice would increase the inequalities that exist between patients from different socioeconomic and geographic areas.^27^ In fact, a retrospective observational study using the Flatiron Health database, which includes longitudinal data of patients with advanced/metastatic solid tumors, revealed that the use of NGS-based biomarkers was the most relevant difference between white and black patients in the United States, impacting the choices of the latter for being recruited in clinical trials.^28^ The application of improved risk models based on simple variables, such as the one proposed in this article, provides a possibility to equalize the access to effective risk stratification tools for most socioeconomic and geographical areas, thereby reducing the disparities associated with the rapid expansion of genomic tools. Nevertheless, if available, an NGS analysis should always be considered due to the following reasons: (1) to improve the quality of the diagnosis; (2) to test for potential drug targets and/or available trials testing targeted drugs; (3) to compare the performance of the different modeling strategies; and (4) to evaluate possible discrepancies and/or complementarities between the different scores.

The main limitations of the present study derive from its registry-based nature. Data quality depends on local physicians entering data at many different centers over a long follow-up period. In this regard, the GESMD group has implemented a centralized review of the data by a hematologist, which contributes to minimizing errors. Nonetheless, the large size of the patient series, followed over a long observation period and the external validation, is a principal strength of our study, reflecting the actual clinical course of the entire MDS population without the set of inclusion and exclusion criteria used in controlled clinical trials. It should also be noted that, contrary to molecular risk models, the present score is intended to be calculated with complete, but easily obtainable, data. Working with complete data is preferable to using imputed data as it ensures more robust and reliable predictions without introducing additional uncertainty from the imputation process. A common criticism of all current prognostic scores relies on the impact of active treatments (e.g., hypomethylating agents) on patient survival. It has been argued that the impact of treatment on survival is minimal because it only affects a minority of high-risk patients, and that external validation in different cohorts should be sufficient to confirm the robustness of the predictor.^24^ At the same time, it has been noted that the exclusion of patients who were subsequently treated for their MDS would represent a bias in the data, with over- and under-optimistic outcome predictions for patients with lower and higher risk disease.^5^ A focused analysis of patients who require active treatment for their MDS is needed to identify optimal treatment strategies.^29^

In conclusion, we present a new prognostic model for MDS based on data from the Spanish Myelodysplastic Syndromes registry. The present model can provide patient-specific predictions, outperforms other well-established risk stratifying systems (e.g., IPSS-R), and has similar accuracy as the IPSS-Mol for the prediction of mortality. Furthermore, this model does not require molecular data, thereby facilitating its applicability in most healthcare settings. An interactive web calculator of the model can be accessed using the following link: https://www.gesmd.es/aipss-mds/

## AUTHOR CONTRIBUTIONS

The GESMD group collected the data. AMO analyzed the data. AMO, MMPE, and DV wrote the article. DC created the online calculator. The remaining authors reviewed the article, made recommendations, and gave final approval for publication.

## DATA AVAILABILITY STATEMENT

The data supporting the findings of this study are not publicly available due to privacy or ethical restrictions but are available on request from the manuscript corresponding authors. The study was approved by the scientific board of GESMD.

## DISCLOSURES

The authors have no conflicts of interest to disclose.

## SOURCES OF FUNDING

The authors report no sources of funding to declare.

## Supplementary Material


