# Supplementary material for: Machine Learning Improves Risk Stratification in Myelodysplastic Neoplasms: An Analysis of the Spanish Group of Myelodysplastic Syndromes
Source: Hemasphere. 2023 Oct 11;7(10):e961. doi: 10.1097/HS9.0000000000000961 (PMC10569758; doi:10.1097/HS9.0000000000000961)
Supplement: Supplementary file 1 [file hs9-7-e961-s001.pdf]

Supplementary Table 1. Proportion of missing data for each variable in the training and test sets.

Variable	Training set	Test set
**Age**	0%	0%
**Gender**	0%	0%
**Peripheral Blood Blasts**	0%	0%
**Bone Marrow Blasts**	0%	0%
**% Nucleated Red Cells in Bone Marrow Smear**	12.5%	11.3%
**Presence of Auer Rods**	35.5%	34.5%
**% of Ring Sideroblasts**	20.7%	19.7%
**Bone Marrow Smear Cellularity**	19.4%	19.3%
**LDH value**	17.3%	18.4%
**Serum Erythopoietin**	56.3%	54.9%
**Free Transferrin Light Chain**	22.5%	22.7%
**Hemoglobin**	0%	0%
**Leukocytes**	0%	0%
**Platelets**	0%	0%
**Neutrophil counts**	0%	0%
**Neutrophil %**	0%	0%
**Monocyte %**	0%	0%
**Karyotype**	5.3%	6.0%
