# Supplementary figures and images for: Machine Learning Improves Risk Stratification in Myelodysplastic Neoplasms: An Analysis of the Spanish Group of Myelodysplastic Syndromes
Source: Hemasphere. 2023 Oct 11;7(10):e961. doi: 10.1097/HS9.0000000000000961 (PMC10569758; doi:10.1097/HS9.0000000000000961)

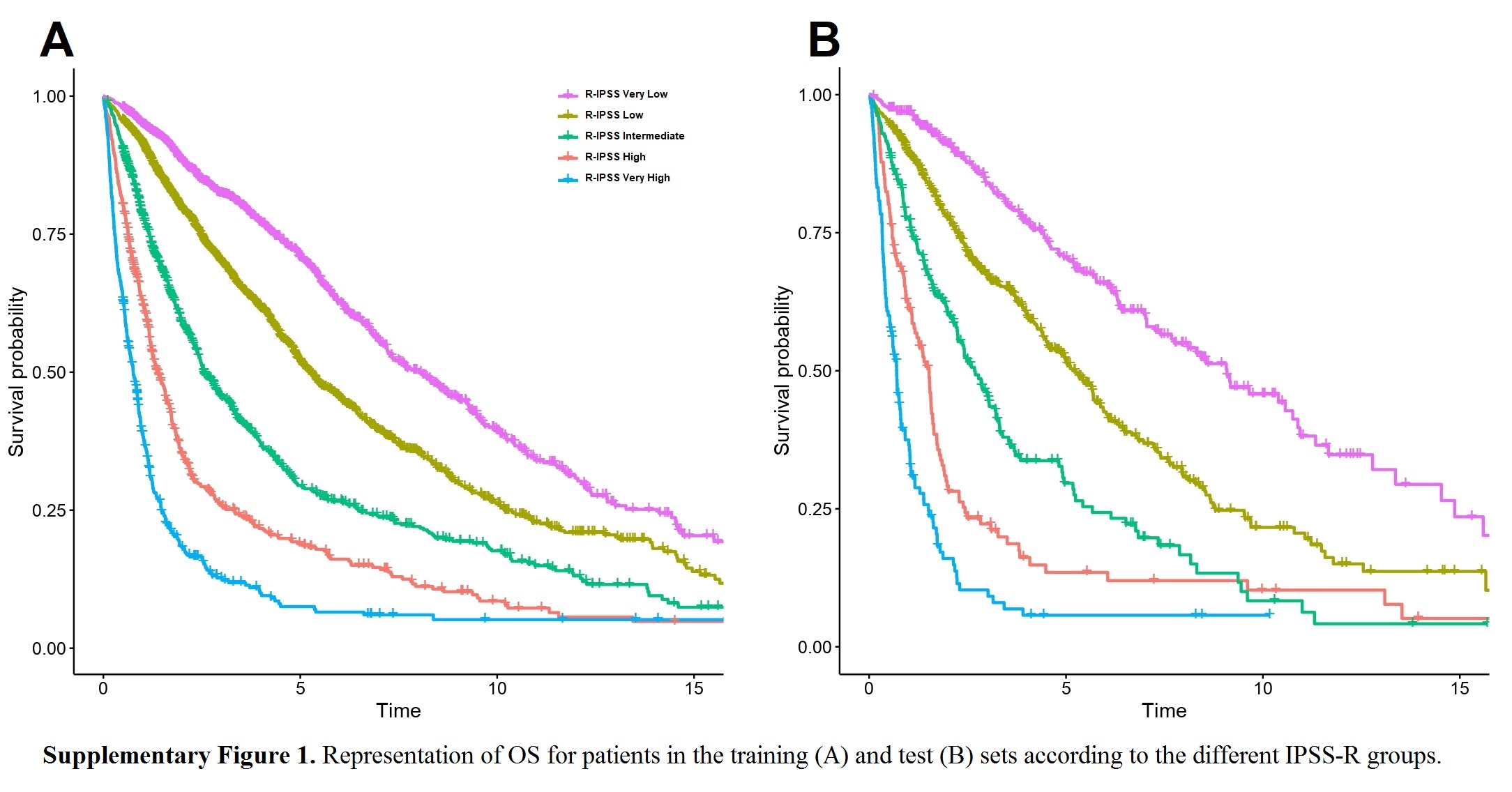

Supplement: Supplementary file 2 [file hs9-7-e961-s002.jpg]

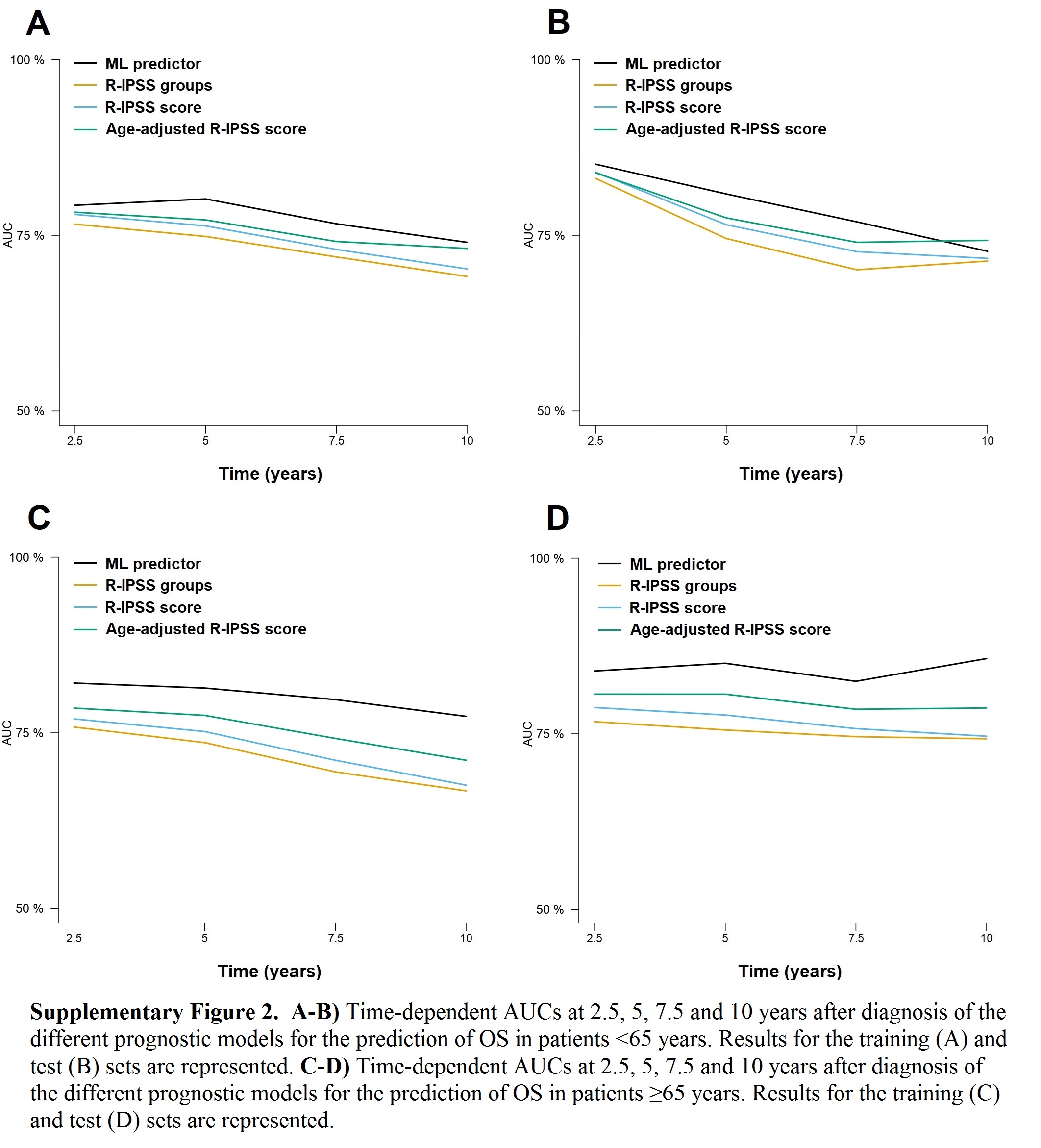

Supplement: Supplementary file 6 [file hs9-7-e961-s006.jpg]

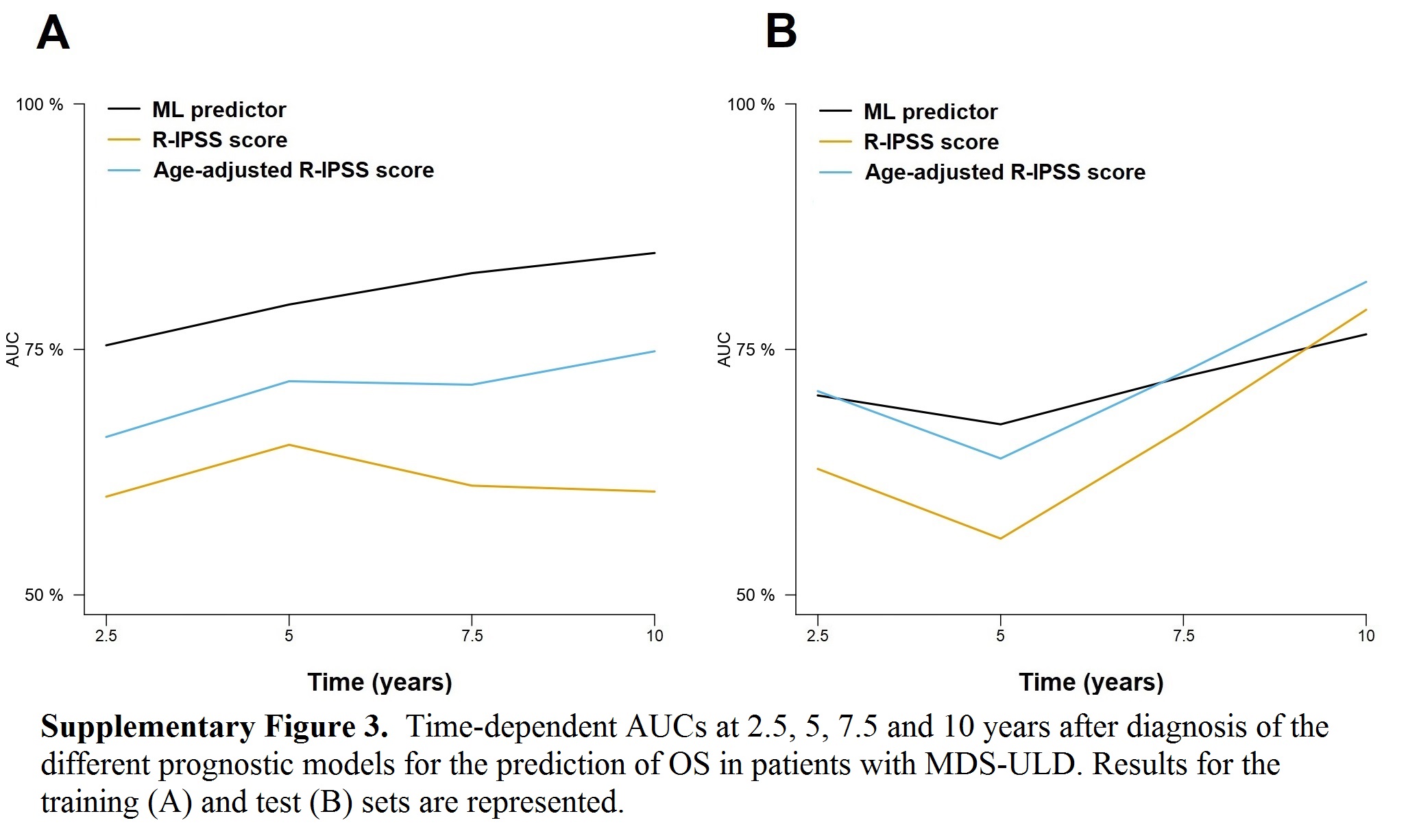

Supplement: Supplementary file 8 [file hs9-7-e961-s008.jpg]

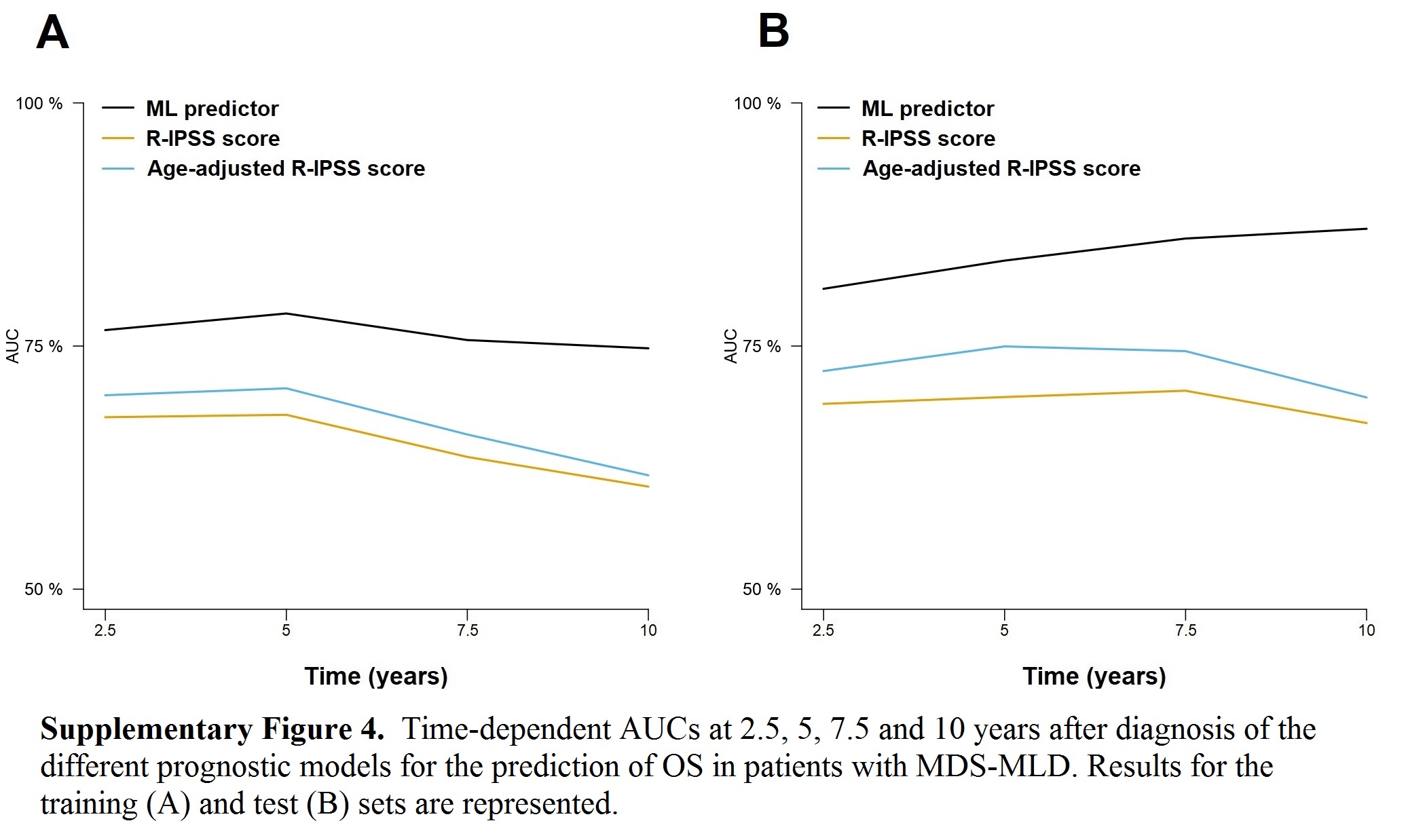

Supplement: Supplementary file 9 [file hs9-7-e961-s009.jpg]

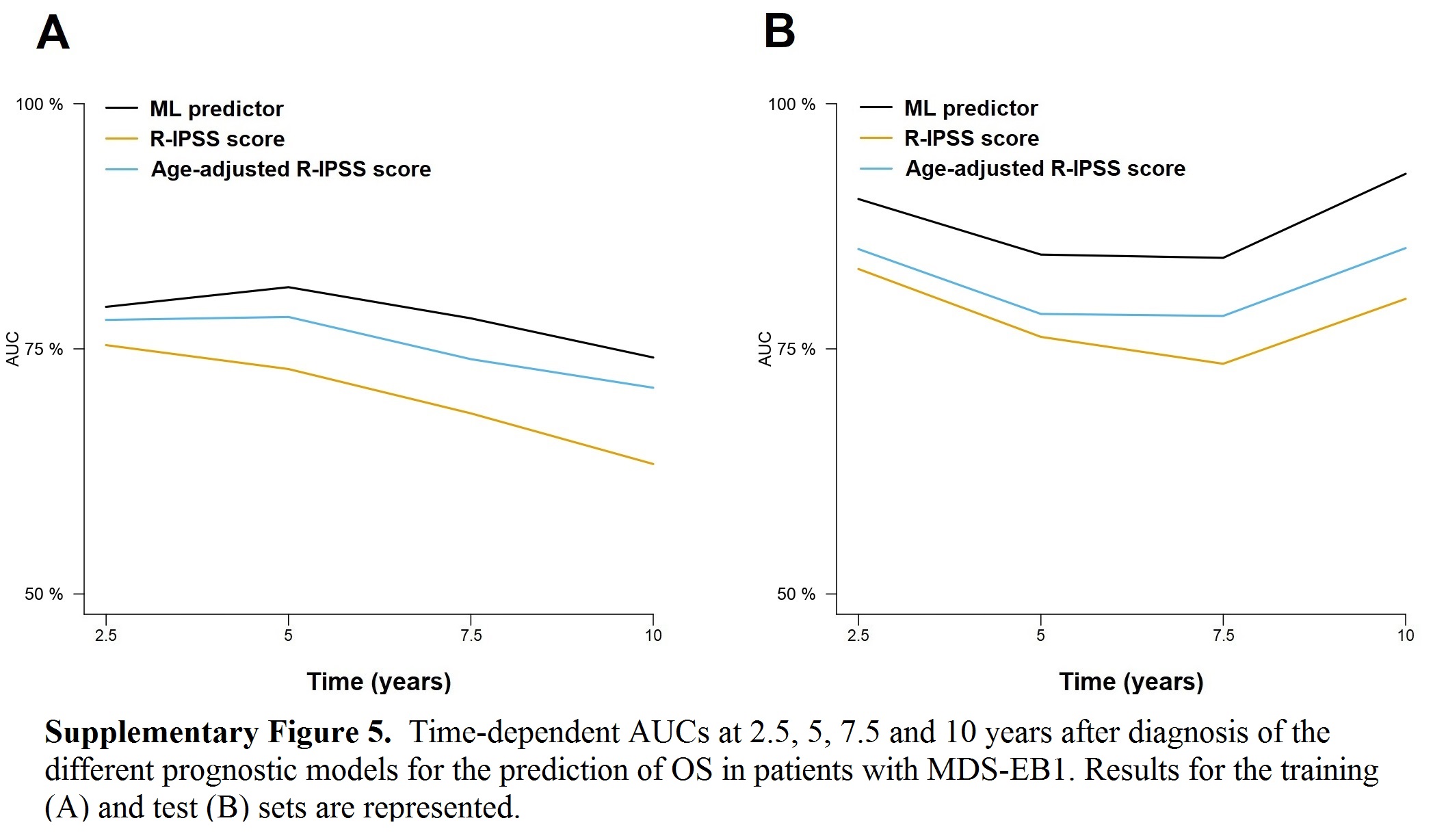

Supplement: Supplementary file 10 [file hs9-7-e961-s010.jpg]

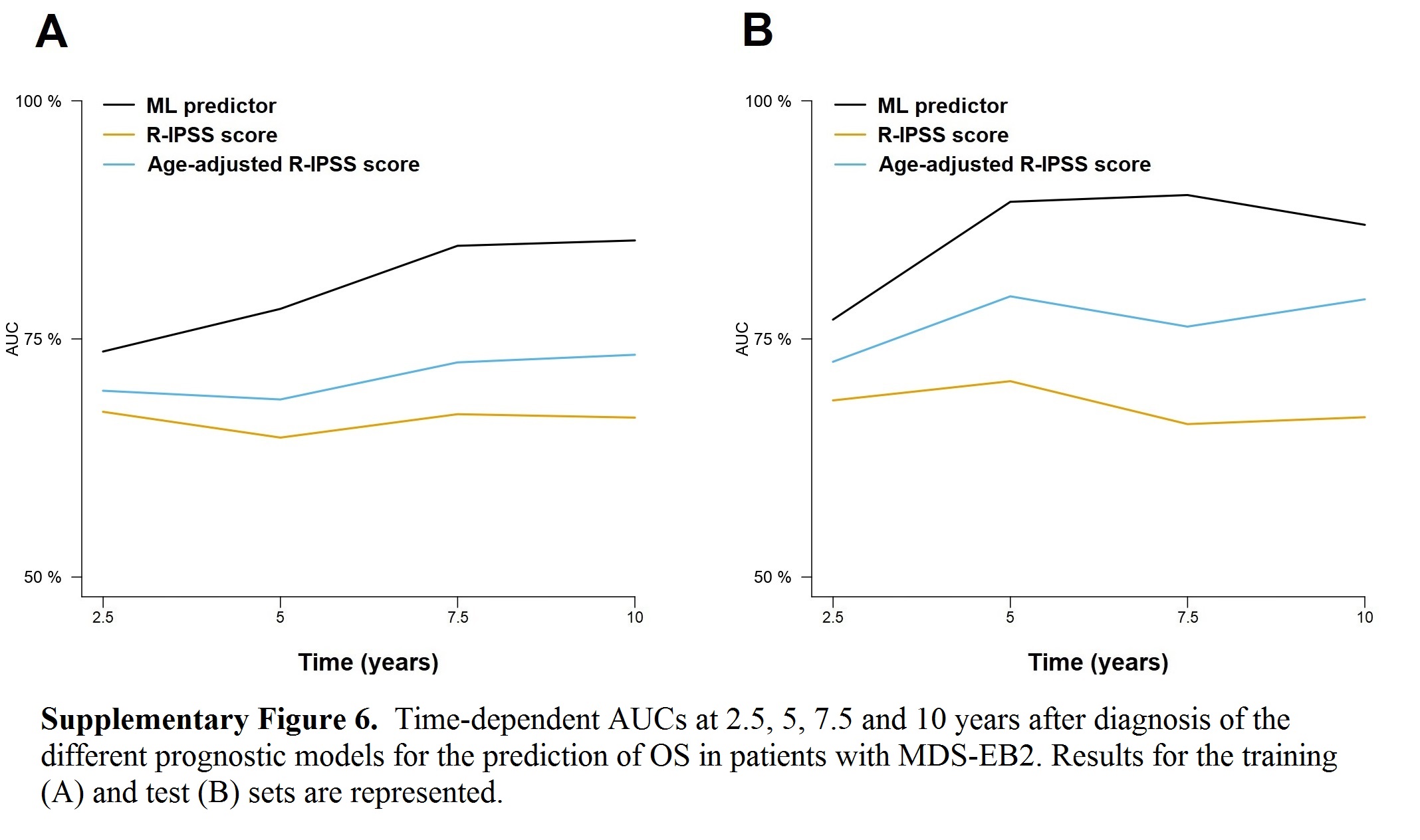

Supplement: Supplementary file 11 [file hs9-7-e961-s011.jpg]

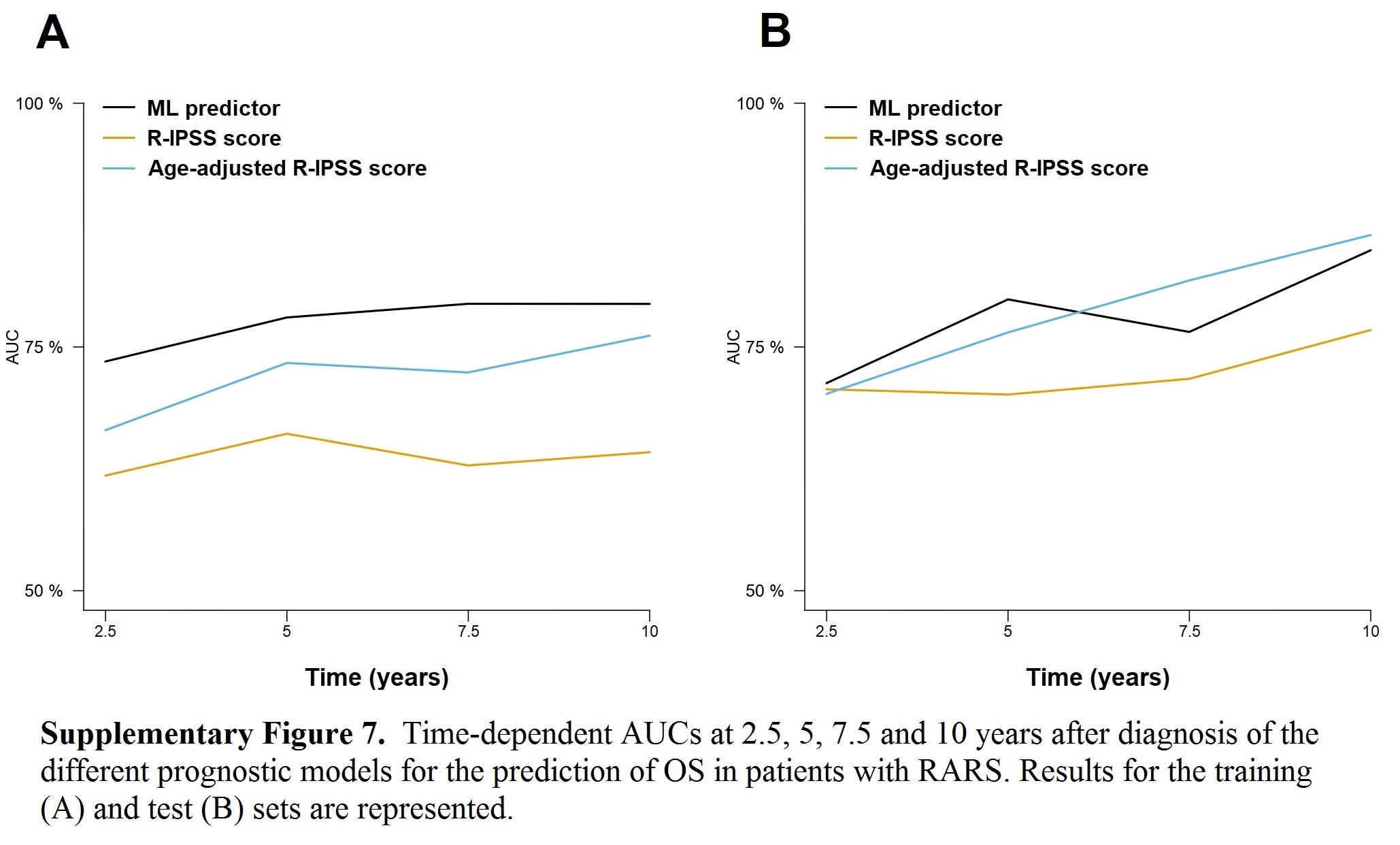

Supplement: Supplementary file 12 [file hs9-7-e961-s012.jpg]

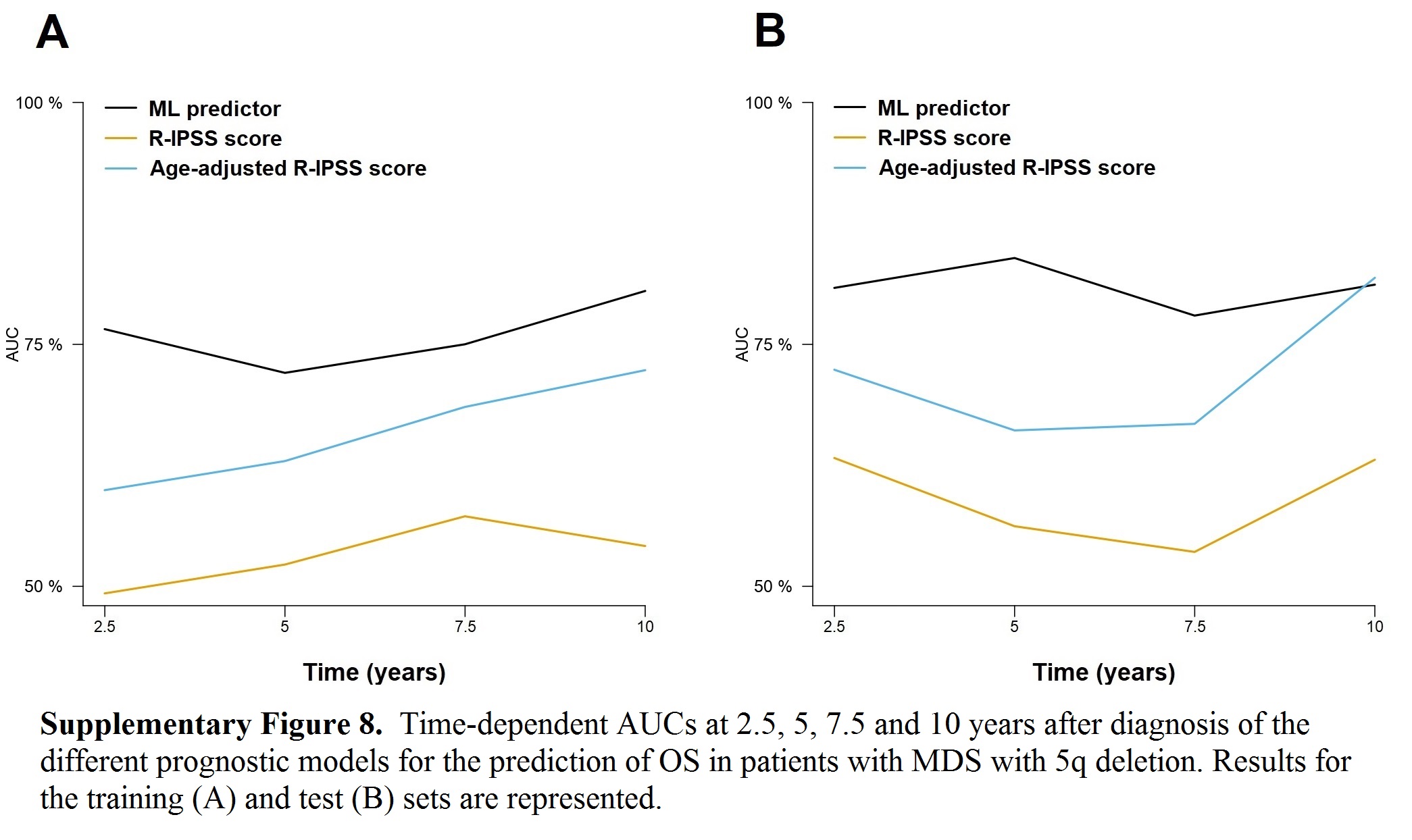

Supplement: Supplementary file 13 [file hs9-7-e961-s013.jpg]
